# A Case of Plasmodium falciparum Malaria with a Prolonged Incubation Period of Four Years

**DOI:** 10.7759/cureus.7176

**Published:** 2020-03-04

**Authors:** Elsa Sleiman, Astha Upadhyaya, Allison Glaser, Michael Krzyzak

**Affiliations:** 1 Internal Medicine, Northwell Health-Staten Island University Hospital, New York, USA; 2 Internal Medicine, Odessa National Medical University, Odessa, UKR

**Keywords:** malaria, chemoprophylaxis

## Abstract

With increased global travel, unique diseases from different parts of the world pose a new category of differential diagnosis, and an incubation period of different infectious diseases helps narrow differential diagnosis to point to one specific etiology. The normal incubation period of *Plasmodium falciparum *malariais 7-40 days. We report a case of symptomatic *P. falciparum* malaria that manifested four years after a visit to a malaria-endemic area in a 51-year-old female patient. Our case illustrates the importance of a broad differential with regard to infectious disease including remote travel history and raises questions of the survival of *P. falciparum* for four years despite the administration of traditional chemoprophylactic agents.

## Introduction

Malaria is a mosquito-borne disease, transmitted by the Anopheles mosquito [1]. The normal incubation period of *Plasmodium falciparum* malaria is 7-40 days, unlike other species such as *P. vivax* and *P. ovale*, which may remain dormant in the liver for up to about four years. It is also responsible for the most serious form of the disease [2]. However, in cases of partial immunity, sickle cell disease or a history of chemoprophylaxis, the incubation period can become prolonged and can lead to a late presentation of clinical symptoms from weeks to months. If not diagnosed early and treated adequately, *P. falciparum *malaria can lead to life-threatening complications, such as shock, lactic acidosis, seizures, and renal failure. The underlying pathology is explained by the visible clumping of the erythrocytes in deep capillary beds. We report a case of symptomatic *P. falciparum* malaria that manifested four years after a visit to a malaria-endemic area in a 51-year-old woman. This patient lives in the United States and had received appropriate malaria chemoprophylaxis four years prior to presentation. This surprisingly long incubation period raises questions regarding the survival and fitness of *P. falciparum* prior to disease manifestations and the rising resistance to traditional chemoprophylactic agents.

## Case presentation

Clinical presentation

A 51-year-old female from Liberia with a past medical history of hypertension, gastroesophageal reflux, and appendectomy presented to the emergency department with fever, generalized body aches, malaise, and headache for two days. In the emergency department, she was found to have a fever of 101.3 °F and tachycardia, with normal blood pressure and respiratory status. She reported no other symptoms. Her last visit to Liberia was four years ago. She denied any complications after her travel, including fever, fatigue, and anemia. The patient reported chemoprophylaxis but was unable to remember the regimen. She reported no travel outside of the New York area since then, including airports. She denied any blood transfusions or any exposure to wooded areas or tick bites. She worked as a home health aide; she denied smoking, alcohol, or illicit drug use.

**Figure 1:**
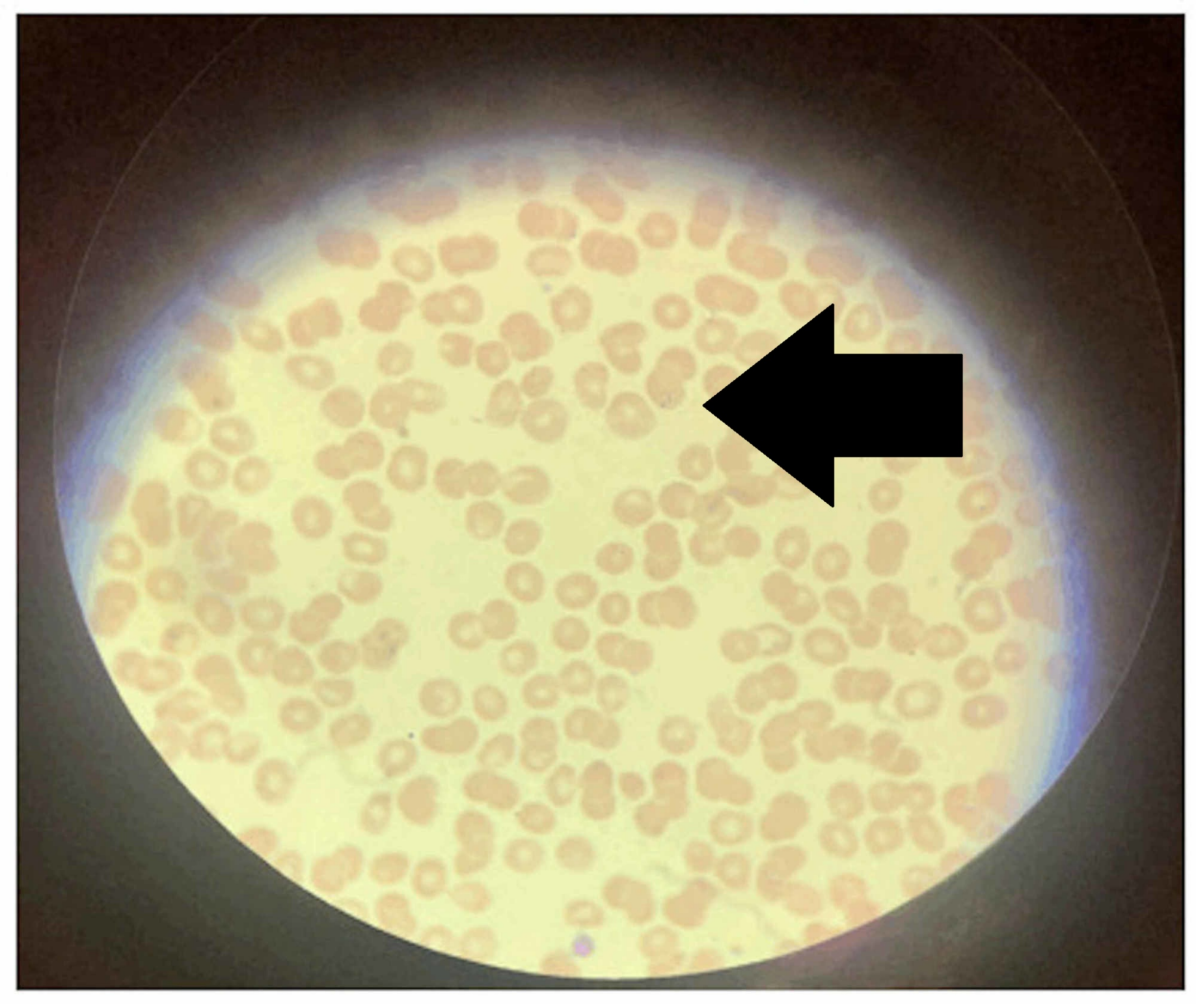
Double dotted ring gametocytes shown by an arrow inside a red blood cell - typical for Plasmodium falciparum infection

Her laboratory tests revealed thrombocytopenia with a platelet count of 32,000 per microliter (normal: 130,000-400,000 per microliter), leukopenia with a white blood cell count of 3,200 per cubic milliliter (normal: 4,800-10,800 per cubic milliliter), and anemia with a hemoglobin of 9.2 g/dL (normal: 12.0-16.0 g/dL). Lactate dehydrogenase was 310 IU/L (normal: 50-242 IU/L) and bilirubin 3.8 mg/dL (normal: 0.2-1.2 mg/dL), mostly indirect. Labs revealed elevated liver function enzymes with alanine aminotransferase (ALT) and aspartate aminotransferase (AST) being 57 and 67 U/L, respectively (normal: <41 U/L). Ultrasound of the liver was normal. Peripheral blood smear showed *P. falciparum* on Giemsa stain with a parasitemia of 1.86% (normal <1%; Figure 1). Molecular detection of *Plasmodium *using polymerase chain reaction (PCR) yielded positive results. A diagnosis of malaria with *Plasmodium falciparum* was made.

Imaging

Chest X-ray was performed and was negative for any focal parenchymal opacities, pleural effusions, or pneumothorax. No splenomegaly or hepatomegaly was evident on computed tomography (CT) abdomen/ pelvis or abdominal ultrasound.

Treatment and management

The patient was treated with Malarone (atovaquone 1000 mg/proguanil 400 mg) once daily for 4 consecutive days. A decline in parasitemia was confirmed as the patient responded to treatment; the last smear showed <1% parasitemia. Thrombocytes, lymphocytes, and lactate dehydrogenase were closely monitored until they normalized. The patient was discharged home with outpatient follow-up with an infectious disease specialist.

## Discussion

There are five pathogenic species of Plasmodium causing malaria in humans: *P. vivax, P. falciparum, P. malariae, P. ovale, *and* Plasmodium knowlesi. P. vivax *and *P. falciparum* are the two most common species found in Africa. *P. falciparum *is the most lethal of all. The life cycle of malaria parasites begins when an infected female Anopheles mosquito bites an uninfected individual. Parasites stick to red blood cells and clump deformed erythrocytes in deep capillary beds, predisposing to organ ischemia and shock, as well as tissue hypoxia and lactic acidosis.

A total of 2.4 billion people are at risk of stable or unstable *Pl.falciparum* transmission with 350 to 500 million clinical episodes and 1 million deaths annually [1]. According to the Center for Disease Control, malaria can present after weeks to months after a history of travel to malaria-endemic regions in the presence of chemoprophylaxis, especially in cases of non-falciparum malaria species such as *P. vivax *and *P. ovale*. The normal incubation period for malaria is 7-40 days. It is shorter for falciparum species compared to non-falciparum species such as *P. vivax* and *P. ovale *[3]. Chemoprophylaxis or partial immunity to malaria can prolong this incubation period [4]. The longest incubation period found on a literature search of *P. falciparum* was eight years after visiting an endemic area [5]. Our case report suggests that *P. falciparum* malaria can present even after four years in a patient with a remote history of travel to an endemic region, in spite of receiving chemoprophylaxis.

Repeated parasite exposure can lead to clinical immunity initially protecting from severe consequences. However, as transmission rates decrease, the risk of infection also increases the rates of severe disease due to the lack of functional immunity [6]. Outbreaks have been reported with *P. falciparum* with reported infected United States marines in Liberia in 2003 [7]. In obtaining histories from patients, it is important to keep in mind remote travel histories as they can indicate potential exposures as illustrated in this case.

## Conclusions

From this case report, we conclude that our patient had *P. falciparum* malaria that she acquired in Liberia four years prior to her clinical presentation. As *P. falciparum* malaria is a fatal disease, it is crucial to have a high index of suspicion and investigate for disease in patients presenting with fever, malaise, and thrombocytopenia, even with a remote history of travel to an endemic region. Early appropriate management in these patients can prevent fatal complications and even death.
